# Supplementary material for: EMT-related gene expression is positively correlated with immunity and may be derived from stromal cells in osteosarcoma
Source: PeerJ. 2020 Feb 3;8:e8489. doi: 10.7717/peerj.8489 (PMC7003688; doi:10.7717/peerj.8489)
Supplement: Supplemental Information 3 [file peerj-08-8489-s003.docx]

**Supplementary materials**

**Table 1 The primer sequence of the EMT-related genes**

| **Genes** | **Primer forward** | **Primer reverse** |
| --- | --- | --- |
| ABI3BP | CAAATGCAACATGCTCTCCAGT | TTGGCCTTTTACCTTTTGGCA |
| AREG | GTGGTGCTGTCGCTCTTGATA | CCCCAGAAAATGGTTCACGCT |
| CADM1 | ATGGCGAGTGTAGTGCTGC | GATCACTGTCACGTCTTTCGT |
| CDH6 | AGAACTTACCGCTACTTCTTGC | TGCCCACATACTGATAATCGGA |
| COL1A1 | GAGGGCCAAGACGAAGACATC | CAGATCACGTCATCGCACAAC |
| COL4A2 | TTATGCACTGCCTAAAGAGGAGC | CCCTTAACTCCGTAGAAACCAAG |
| FGF2 | AGAAGAGCGACCCTCACATCA | CGGTTAGCACACACTCCTTTG |
| GPX7 | CCCACCACTTTAACGTGCTC | GGCAAAGCTCTCAATCTCCTT |
| LAMA2 | TGCTGTCCTGAATCTTGCTTC | AGCATTTGTAATCGGGTGTCTC |
| LEPRE1 | CAAGCGGAGCCCCTACAAC | CTCCCAGTCGAAATTCTTGCAT |
| MATN3 | TCTCCCGGATAATCGACACTC | CAAGGGTGTGATTCGACCCA |
| SERPINE2 | TGGTGATGAGATACGGCGTAA | GTTAGCCACTGTCACAATGTCTT |
| SERPINH1 | TCAGTGAGCTTCGCTGATGAC | CATGGCGTTGACTAGCAGGG |
| SNAI2 | CGAACTGGACACACATACAGTG | CTGAGGATCTCTGGTTGTGGT |
| SPARC | TGAGGTATCTGTGGGAGCTAATC | CCTTGCCGTGTTTGCAGTG |
| THY1 | ATCGCTCTCCTGCTAACAGTC | CTCGTACTGGATGGGTGAACT |
| VCAN | GTAACCCATGCGCTACATAAAGT | GGCAAAGTAGGCATCGTTGAAA |
| VIM | GACGCCATCAACACCGAGTT | CTTTGTCGTTGGTTAGCTGGT |
| COMP | GATCACGTTCCTGAAAAACACG | GCTCTCCGTCTGGATGCAG |
| GREM1 | CGGAGCGCAAATACCTGAAG | GGTTGATGATGGTGCGACTGT |
| MMP3 | AGTCTTCCAATCCTACTGTTGCT | TCCCCGTCACCTCCAATCC |
| SGCD | GCGGAAACGATGCCTGTATTT | TGGCGTAGAGAGGTTGTAAGAA |
| CD86 | CTGCTCATCTATACACGGTTACC | GGAAACGTCGTACAGTTCTGTG |
| IL18R1 | CCTTGACCCTTTGGGTGCTTA | CTCATGTGCAAGTGAACACGA |
| SPON1 | CCCAAGTCAGAGGGCTACTG | GGTTCCCGGCTTGTAGAAGT |
