# Supplementary material for: EMT-related gene expression is positively correlated with immunity and may be derived from stromal cells in osteosarcoma
Source: PeerJ. 2020 Feb 3;8:e8489. doi: 10.7717/peerj.8489 (PMC7003688; doi:10.7717/peerj.8489)
Supplement: Supplemental Information 4 [file peerj-08-8489-s004.docx]

**Supplementary materials**

**Table 2 The 200 EMT-related genes obtained from the MSigDB**

| List of HALLMARK_EPITHELIAL_MESENCHYMAL_TRANSITION |
| --- |
| COL3A1，COL5A2，COL5A1，FBN1，COL1A1，FN1，COL6A3，SERPINE1，COL1A2，COL4A1，COL4A2，VCAN，IGFBP3，TGFBI，SPARC，LUM，LAMC1，LOX，LAMC2，CTGF，TAGLN，COL7A1，LOXL2，COL6A2，ITGAV，THBS2，COL16A1，NNMT，TPM1，CDH2，MMP2，COL11A1，THBS1，FAP，BGN，SERPINH1，FSTL1，POSTN，THY1，SPP1，TNC，TFPI2，NID2，ITGB5，MMP3，VIM，LOXL1，FBLN5，COL12A1，ELN，CDH11，COMP，SPOCK1，BMP1，IL32，LAMA3，TIMP1，QSOX1，TIMP3，VCAM1，CYR61，EDIL3，CALD1，MAGEE1，FBLN1，SGCB，ECM1，LAMA2，FSTL3，TPM2，INHBA，DAB2，EMP3，BASP1，ITGA5，MGP，VEGFA，CXCL1，WNT5A，SDC1，PLOD2，PCOLCE，GREM1，ITGB1，COL5A3，RHOB，HTRA1，FGF2，SNTB1，GADD45A，MEST，LRRC15，TNFRSF11B，CD59，ACTA2，EFEMP2，MATN2，PCOLCE2，SERPINE2，GPC1，ABI3BP，FUCA1，SLIT3，LAMA1，PMEPA1，COL8A2，FBN2，IGFBP2，PFN2，SDC4，CD44，GADD45B，IL8，GLIPR1，ANPEP，LEPRE1，VEGFC，MMP14，SGCD，PLOD1，MATN3，MYL9，SLC6A8，CALU，PRRX1，TNFRSF12A，FMOD，ID2，GEM，PLAUR，MYLK，TGFB1，SFRP1，PLOD3，IL6，APLP1，FBLN2，MSX1，PTX3，FZD8，JUN，FERMT2，DKK1，SNAI2，DST，TPM4，DCN，GJA1，PMP22，IGFBP4，COPA，LRP1，ITGA2，FLNA，MFAP5，PTHLH，TGFBR3，SFRP4，LGALS1，RGS4，CDH6，SAT1，NT5E，DPYSL3，PPIB，TGM2，SGCG，ITGB3，PDLIM4，CTHRC1，ECM2，CRLF1，AREG，IL15，MCM7，GAS1，PRSS2，CADM1，OXTR，SCG2，CXCL6，MMP1，TNFAIP3，CAPG，CAP2，MXRA5，FOXC2，NTM，ENO2，FAS，BDNF，ADAM12，PVR，CXCL12，PDGFRB，SLIT2，NOTCH2，GLT25D1，GPX7，WIPF1 |
